# Salvage Reconstruction of Composite Defects of the Anterior Mandible, Floor of Mouth, and Lip

**DOI:** 10.1055/a-2263-8046

**Published:** 2024-04-10

**Authors:** Farrukh A. Khalid, Omar A. Ahmed, Almeotan P. Khurshid, Abdul M. Mujahid, Junaid Ahmad, Muhammad Saleem, Muhammad A. Yousaf, Moazzam N. Tarar, Farooq Shahzad

**Affiliations:** 1 Jinnah Burn and Reconstructive Surgery Center, Allama Iqbal Medical College, Lahore, Pakistan; 2 Department of Plastic and Reconstructive Surgery, Royal Victoria Infirmary, Newcastle Upon Tyne, United Kingdom; 3 Plastic and Reconstructive Surgery Service, Memorial Sloan Kettering Cancer Center, New York, New York

**Keywords:** Andy Gump, anterior mandible, free flap, functional intraoral Glasgow scale, lip suspension

## Abstract

Anterior mandible defects result in loss of support for the tongue, floor of the mouth and lower lip, resulting in impairment of airway, feeding, and speech. We treated four patients with these “Andy Gump” deformities. Reconstruction was performed with two free flaps: a fibula osteocutaneous flap for the anterior mandible and floor of the mouth, and a soft tissue free flap for the lip, chin, and anterior neck. The lower lip was suspended cranially with fascia or tendon grafts ± mini-temporalis turndown flaps. All flaps survived completely. All patients were tube feed-dependent before surgery; they all resumed an oral diet. All tracheostomies were decannulated. Lip competence was restored as evidenced by cessation of drooling. Speech improved from unintelligible to intelligible with frequent repetitions. Objective assessment was performed with the functional intraoral Glasgow scale; the mean FIGS score improved from 3.25 (range 3–4) to 11 (range 9–13). We conclude that composite anterior mandible and tongue defects have large tissue requirements that require multiple free flaps. Reconstruction leads to significant improvement in function.

## Introduction

The anterior mandible plays a crucial role in orofacial function, serving as a point of attachment for the tongue, floor of the mouth, and the lower lip. Unrepaired defects of the mandibular arch result in tongue prolapse and lip incompetence, leading to problems with the airway, speech, feeding, and salivary control. The aesthetic deficit from loss of chin projection is called the “Andy Gump” deformity. 1

Composite defects of the anterior mandible, floor of mouth, and lower lip pose a reconstructive challenge, as multiple tissue types and surfaces need to be restored. When these defects are encountered in a secondary manner, the complexity of the reconstruction is significantly increased; tissue contraction distorts the normal anatomy, making clinical assessment difficult. Often these patients are oral cripples who are dependent on feeding tubes and tracheotomies. Abnormal physical appearance can have devastating psychosocial consequences.

Reconstruction of these defects is complex and frequently requires multiple free flaps for bone and soft tissue reconstructions. As one of the few microsurgery centers in a low-resource country, we have encountered many of these unfortunate patients. This article presents our experience with the treatment of these defects with the goal of providing useful insights into how these patients may be managed.

## Case

This is a retrospective case series of patients with composite defects comprising the anterior mandible and the adjacent floor of mouth and lower lip, who presented in a delayed fashion and underwent free flap reconstruction. All patients had either previous failed attempts at reconstruction or had no reconstruction performed. Informed consent was obtained from all patients, including the use of their full-face pictures.

Preoperative workup included orthopantomography and computed tomography of the face. Patients either had preexisting tracheostomies or had undergone surgery at the time of surgery. Reconstruction was performed with two free flaps, a fibula osteocutaneous flap for mandible and floor of mouth reconstruction, and a soft tissue free flap for lip, chin, and anterior neck reconstruction. The lower lip was suspended cranially with either tendon or fascia lata sling.

All patients were followed up for a minimum of 3 years. Functional assessment was performed using the functional intraoral Glasgow scale (FIGS; Table 1 ). 2 3 This validated five-point scale assesses chewing, swallowing, and speech to provide a global disability score from 3 (worst) to 15 (best). 4 FIGS scores were obtained preoperatively and at the last follow-up.

**Table 1:** Functional intraoral Glasgow scale 3 : a score from 1 to 5 is assigned to each category for a global score between 3 and 15

Score	Chewing	Swallowing	Speech
5	Any food without difficulty	Any food without difficulty	Always clearly understood
4	Solid food with difficulty	Solid food with difficulty	Repetition required sometimes
3	Semisolid food without difficulty	Semisolids	Repetition required many times
2	Semisolid food with difficulty	Liquids	Understood only by relatives
1	Unable to chew	Unable to swallow	Unintelligible

### Surgical Technique

Preoperative nutritional evaluation is performed. These patients are typically undernourished due to severe difficulties with oral intake and a lack of support from trained dieticians. Nasogastric and percutaneous gastric tubes are usually required for nutritional repletion. Labs such as albumin and prealbumin are useful for monitoring nutritional restoration. Surgery is performed once nutrition is optimized.

Tracheostomy is performed at the beginning of the operation. Two teams work simultaneously, one preparing the recipient site and the other harvesting the flap. If available, a third team simultaneously harvests the second flap.

The first step is to recreate the defect. The facial scar is completely excised, and any existing mandibular plates are removed. Tissues are restored to their anatomic position. This usually results in the creation of a large defect. Missing tissues typically include the mandibular symphysis and anterior body, central neck skin, full thickness of the lower lip, anterior floor of the mouth, and occasionally the ventral tongue.

Neck vessels are dissected and prepared. This can be challenging because of the neck fibrosis caused by previous infections and secondary healing. Furthermore, some first-line vessels may have already been used in previous free flap attempts. The unused or lesser used side of the neck is preferred for recipient vessel preparation.

**Fig. 1:**
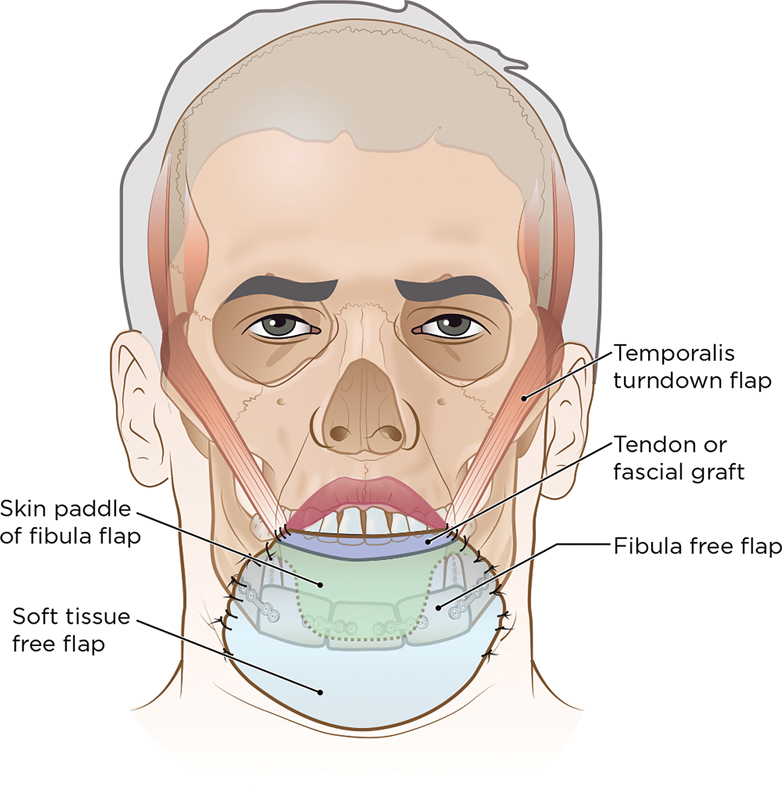
Schematic of the reconstruction. A fibula osteocutaneous flap was used for reconstruction of the mandible, overlying soft tissue, and anterior floor of the mouth. A second soft tissue free flap was used for the reconstruction of the lip, chin, and central neck. The lower lip is suspended cranially with fascia or tendon slings ± mini-temporalis turndown flaps.

The requirements for each missing tissue components are determined. Reconstruction is performed with two free flaps ( Fig. 1 ). A fibula osteocutaneous flap is used for mandible reconstruction and intraoral resurfacing. The fibula skin paddle is used to recreate the inner lip, floor of the mouth, and, if needed, the ventral tongue. The fibula bone is osteotomized into a minimum of three segments to replace the symphysis and each body. The length of bone required and the angle of osteotomies are estimated either by prebending a reconstruction plate around the native mandible or templating a wooden spatula. A 2.0- or 2.4-mm titanium reconstruction plate is bent and fixed to the segments while on the leg. The fibula flap is then detached and inset, with bony plate fixation followed by soft-tissue intraoral inset. Microvascular anastomoses are then performed to vessels in the neck.

A second soft tissue free flap is harvested from either the thigh or the abdomen. This is used for external resurfacing of the lip, chin, and anterior neck. This flap is partially inset and microvascular anastomoses are completed.

Lower lip suspension is then performed. This is a crucial step in preventing oral incontinence. A temporal scalp incision is made on both sides. A strip of temporalis muscle and overlying deep temporal fascia, approximately 3 cm wide, is elevated from the superior temporal line to the zygomatic arch. The deep temporal fascia is then stripped away from the underlying muscle, except cranially, where the fascia is sutured to the muscle to prevent shearing and separation. The muscle and fascia strip is then turned over the zygomatic arch and tunneled in a subcutaneous plane up to the reconstructed lower lip. A rolled fascia lata strip or plantaris tendon graft is passed under the newly created lip to form a sling between both temporalis flaps. Alternatively, lip suspension can be performed without a temporalis flap by harvesting a long strip of fascia lata, tunneling it under the reconstructed lip, and suspending it to the zygoma or deep temporal fascia. The tension is adjusted to achieve maximal vertical lip height and create a lower labial sulcus.

Inset of the soft tissue flap is then completed. The neck is washed out, drains are placed, and final closure is performed. A nasogastric tube is placed if a feeding tube was absent preoperatively.

### Results

Four patients underwent double free flap reconstruction of composite anterior mandible and soft tissue defects ( Table 2 ). There were two males and two females with a mean age of 26.75 years (range 22–30 years). The etiology of the defects was firearm wounds in three patients and a motor vehicle accident in one patient. The time from the original injury to presentation at our center ranged from 3 months to 20 years. All patients were tube feed-dependent.

**Table 2:** Patient data

Patient	Age (years)	Gender	Etiology of defect	Initial reconstruction	Time from injury	Bone flaps used for reconstruction	Soft tissue flap used for recon	Lower lip suspension	Recipient artery	Recipient vein
1	30	Female	Motor vehicle accident	*First attempt* : Pectoralis major flap and reconstruction plate *Second attempt:* Fibula osteocutaneous free flap	12 months	Fibula osteocutaneous free flap	Anteromedial thigh flap	Fascia lata	Facial artery, superior thyroid artery	Internal jugular (end to side)
2	26	Female	Firearm injury	*First attempt:* supraclavicular and pectoralis major flaps *Second attempt:* Anterolateral thigh free flap and reconstruction plate *Third attempt:* Anterolateral thigh free flap	20 years	Fibula osteocutaneous free flap	Vertical rectus abdominis musculocutaneous flap	Plantaris tendon and mini-temporalis turndown flaps	End to end with external carotid artery, facial artery	Internal jugular (end to side)
3	22	Male	Firearm injury	Primary closure of soft tissue	3 months	Fibula osteocutaneous free flap	Anterolateral thigh free flap	Fascia lata and mini-temporalis turndown flaps	Facial artery, superior thyroid artery	Internal jugular (end to side)
4	29	Male	Firearm injury	Pectoralis major flap and reconstruction plate	10 months	Fibula osteocutaneous free flap	Anterolateral thigh free flap	Fascia lata	Facial artery, superior thyroid artery	Internal jugular (end to side)

One patient had a preexisting tracheostomy, while the other three underwent tracheostomy placement at the time of surgery. A fibula osteocutaneous flap was performed in all patients. The second soft tissue flap was an anterolateral thigh flap in two patients, an anteromedial thigh flap in one patient, and a rectus abdominis musculocutaneous flap in one patient. Suspension of the reconstructed lower lip was performed with mini-temporalis turndown flaps plus fascia or tendon slings in two patients, and with fascia lata slings alone in two patients. The mean operative time was 13 hours (range 10–18 h).

All eight flaps survived completely. One minor wound dehiscence healed with local wound care and oral antibiotics. Tracheostomies were decannulated at a mean of 8 days (range 7–10 d). All patients resumed an oral diet and their feeding tubes were removed. One patient had alar rim necrosis due to pressure from the endotracheal tube that healed without intervention. The mean hospital stay was 12 days (range 10–21 d).

The preoperative and postoperative FIGS scores are shown in Table 3 . Chewing scores improved from a mean of 1 (unable to chew) to 3.5 (3, semisolids; 4, solid food with difficulty). Swallowing scores improved from a mean of 1 (unable to swallow) to 3.5 (3, semisolids without difficulty; 4, solid food with difficulty). Speech scores improved from a mean of 1.25 (unintelligible) to 3.75 (3, repetition required many times; 4, repetition required sometimes). The mean overall FIGS score improved from 3.25 (range 3–4) to 11 (range 9–13).

**Table 3:** Preoperative and postoperative functional intraoral Glasgow scale scores

Case number	Preoperative FIGS score	Postoperative FIGS score
1	*Chewing: 1* *Swallowing: 1* *Speech: 1* **Overall: 3**	*Chewing: 3* *Swallowing: 3* *Speech: 3* **Overall: 9**
2	*Chewing: 1* *Swallowing: 1* *Speech: 1* **Overall: 3**	*Chewing: 3* *Swallowing: 3* *Speech: 4* **Overall: 10**
3	*Chewing: 1* *Swallowing: 1* *Speech: 1* **Overall: 3**	*Chewing: 4* *Swallowing: 4* *Speech: 4* **Overall: 12**
4	*Chewing: 1* *Swallowing: 1* *Speech: 2* **Overall: 4**	*Chewing: 4* *Swallowing: 4* *Speech: 4* **Overall: 13**
**Mean**	**Chewing: 1** **Swallowing: 1** **Speech: 1.25** **Overall: 3.25**	**Chewing: 3.5** **Swallowing: 3.5** **Speech: 3.75** **Overall: 11**

Abbreviation: FIGS, functional intraoral Glasgow scale.

All patients regained lip competence and stopped drooling. Patients with mini-temporalis turndown flaps developed dynamic lip movement with jaw clenching. This was especially helpful while eating, as the lip contracted with biting down and reinforced the lip seal.

Revision procedures, including flap debulking and deepening of the lower labial sulcus, were performed in three patients between 1 and 2 years after reconstruction. One patient declined to undergo further surgery.

### Representative Case

A 5-year-old female sustained a gunshot wound to the face, resulting in loss of the anterior mandible, lower lip, chin, part of the tongue, and anterior floor of the mouth. Previous unsuccessful attempts at reconstruction included pedicled supraclavicular and pectoralis major flaps and two failed anterolateral thigh free flaps over a reconstruction plate.

**Fig. 2:**
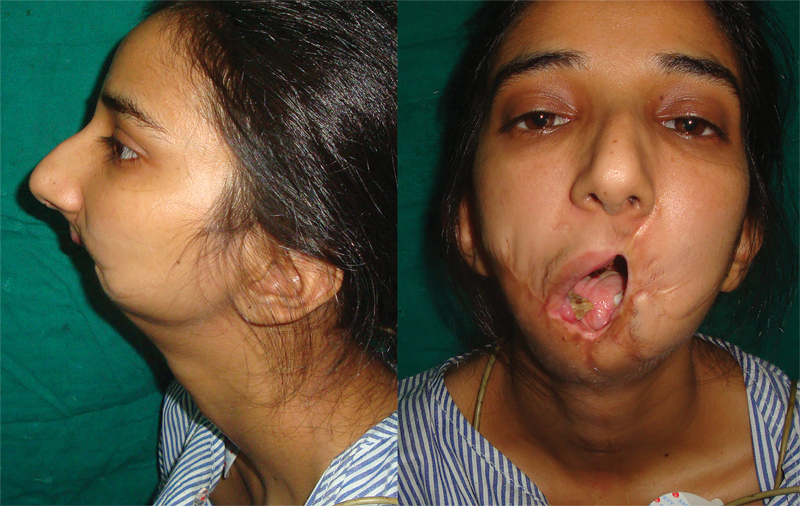
A 26-year-old female who sustained a gunshot wound to the face at the age of 5 years. Previous failed reconstructive attempts included pedicled pectoralis major and supraclavicular flaps and two anterolateral thigh free flaps with a reconstruction plate. Anteroposterior and lateral views showing an Andy Gump deformity.

**Fig. 3:**
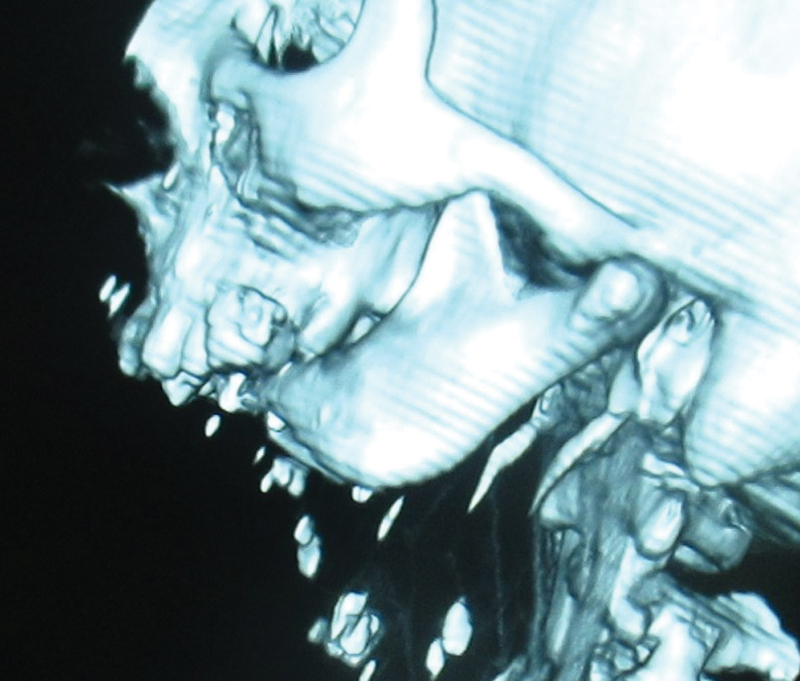
CT scan showing cranial rotation of the mandibular rami.

She presented to our center at the age of 25 years with an Andy Gump deformity, unintelligible speech, constant drooling, tube feeding, and tracheostomy dependence ( Fig. 2 ). CT revealed a very short ascending mandibular ramus on each side, with marked cranial rotation of the remaining mandibular bodies, such that the mandibular remnant was in contact with the hard palate ( Fig. 3 ).

**Fig. 4:**
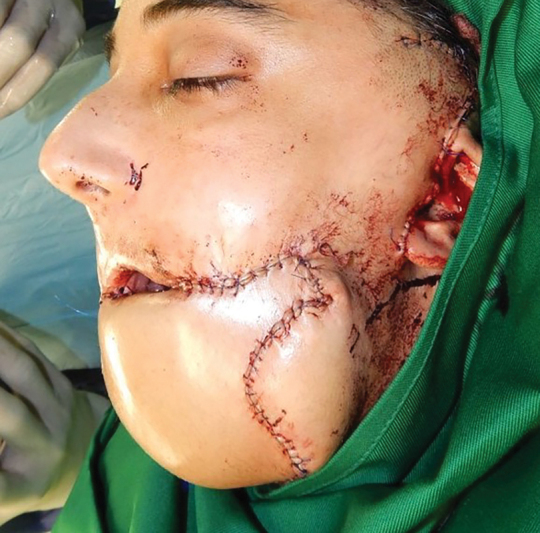
Reconstruction with a fibula osteocutaneous flap and a vertical rectus abdominus musculocutaneous flap.

She was taken to the operating room, and all scars were radically debrided to recreate the defect. The ankylosis of both temporomandibular joints was released, and the deep temporal fascia was placed as a spacer between the condyles and condylar fossae. The anterior mandible, floor of the mouth, and ventral tongue were reconstructed using a fibula osteocutaneous flap. The lower lip, chin, and neck were reconstructed using a vertical rectus abdominis musculocutaneous flap ( Fig. 4 ). Lip suspension was performed using mini-temporalis turndown flaps attached to a plantaris tendon sling.

**Fig. 5:**
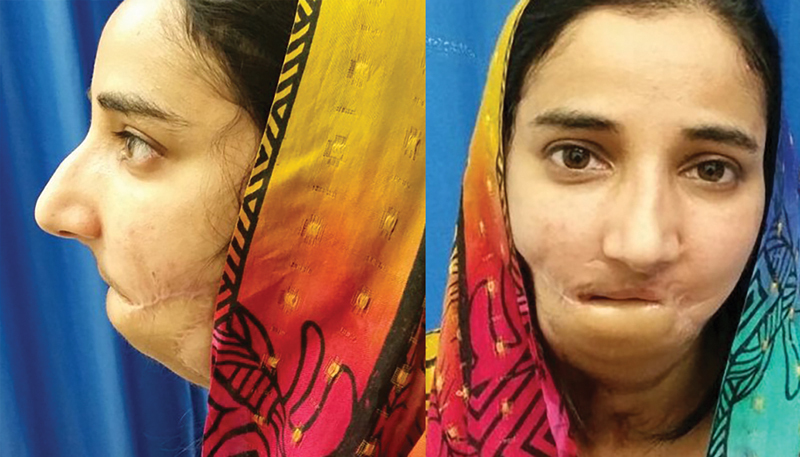
Final result after flap liposuction and vestibuloplasty.

The postoperative course was uneventful. The tracheostomy tube was removed after 10 days, and the nasogastric tube was removed after 5 weeks. Revision procedures were performed 15 and 18 months later, consisting of neck and chin liposuction, and vestibuloplasty, respectively ( Fig. 5 ).

**Fig. 6:**
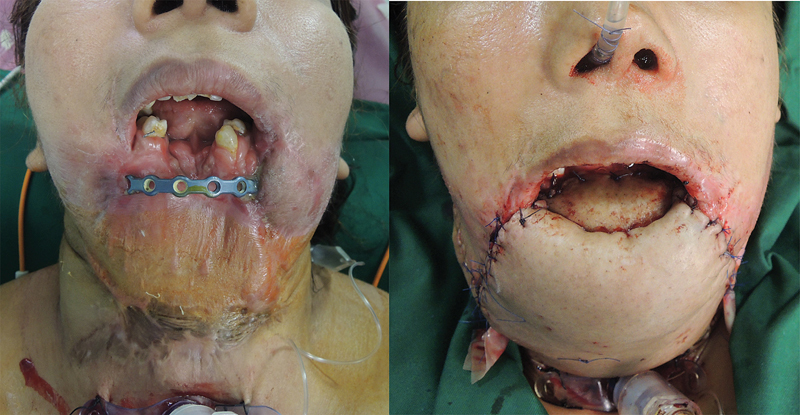
Pre- and postprocedure pictures of a 30-year-old female with through and through anterior mandible loss. Previous attempts at outside institutions included free fibula flap and pedicled pectoralis major flap with reconstruction plate. Definitive reconstruction was performed with fibula osteocutaneous and anteromedial thigh free flaps.

Pre- and postprocedure pictures of the other three patients in this series are shown in Figs. 6 7 8 .

**Fig. 7:**
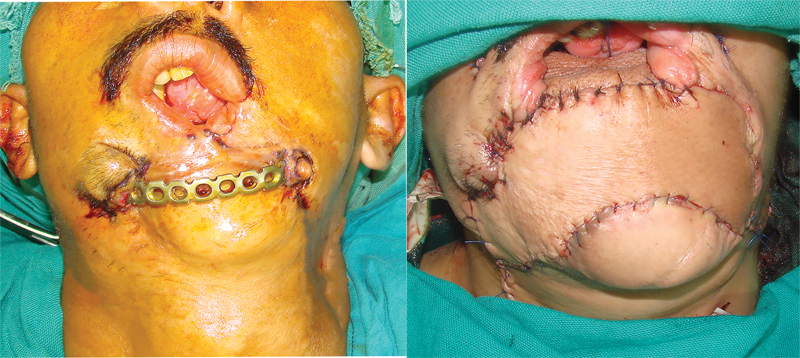
Pre- and postprocedure pictures of a 29-year-old male with through and through anterior mandible loss due to a gunshot wound. He underwent reconstruction at an outside hospital with a pedicled pectoralis major flap and reconstruction bar which was complicated by plate extrusion. Definitive reconstruction performed with fibula osteocutaneous and anterolateral thigh free flaps.

**Fig. 8:**
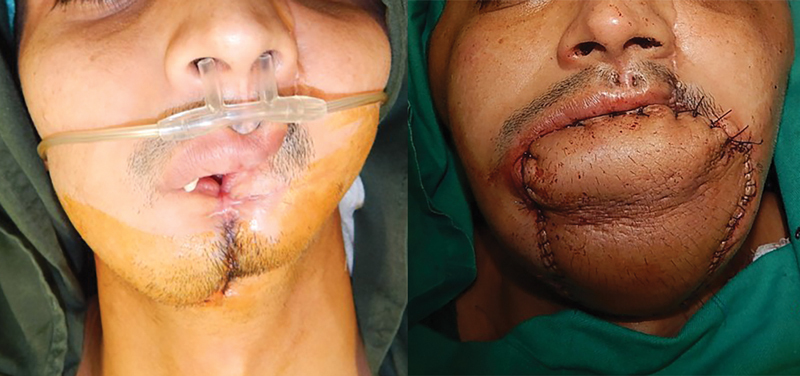
Pre- and postprocedure pictures of a 22-year-old male who sustained a gunshot wound to the mandible, which was temporized at an outside hospital with debridement and primary closure. He presented to our unit 3 months later. Definitive reconstruction was performed with fibula osteocutaneous and anterolateral thigh free flaps.

## Discussion

Acquired composite defects of the anterior mandible can involve the bone, oral lining, external skin, and soft tissues. Trauma and tumor ablation are the most common etiologies. Reconstruction of such composite defects is technically challenging, particularly when reconstructive surgery is attempted months or even years after the original insult or after previous failed attempts. The characteristic physical appearance of loss of lower face projection is known as the “Andy Gump” deformity, named after an American comic strip character from the early 20th century. 1 This deformity is characterized by a segmental defect of the anterior mandible, associated with a variable deficiency of the oral lining and skin. In extreme cases, deficiencies can occur in the chin and lower lip. The loss of the anterior mandibular arch has several functional consequences. The largest muscle of the tongue, the genioglossus, originates from the inner surface of the mandibular symphysis, and is the only muscle that protrudes the tongue forward. Loss of this attachment results in prolapse of the tongue into the pharynx. 5 The mandibular arch also supports the lower lip and is a point of origin for the floor of the mouth musculature. Therefore, these patients can have severe physical disfigurement associated with oral incontinence, difficulty speaking, chewing, swallowing, and airway compromise. Abnormal physical appearance and constant drooling can have major psychosocial consequences.

Delayed reconstruction of composite mandibular defects is challenging because of scarring and wound contractures. Scarring causes distortion of the normal anatomy, making accurate replacement of tissues very difficult. Furthermore, previous reconstructive attempts have led to a paucity of flap donor sites and recipient neck vessels. Through-and-through defects of the anterior mandible are typically reconstructed using one of the following techniques:

1. *Pedicled flap* : A reconstruction plate is used to bridge the bone defect and covered with a pedicled flap like the pectoralis major musculocutaneous flap. 6
2. *One free flap* : Fibula osteocutaneous flap is used for bone reconstruction, with its skin paddle either folded for intra- and extraoral resurfacing or used as two separate skin islands, each with their own perforator. 7 Alternatively, a chimeric scapula osteocutaneous and soft tissue flap can provide a large amount of soft tissue and reasonable bone stock.
3. *Free flap and pedicled flap* : Fibula osteocutaneous free flap is used for bone and intraoral reconstruction and a regional pedicled flap like a pectoralis major musculocutaneous flap or supraclavicular flap is used for external coverage. 8
4. *Two free flaps* : Fibula osteocutaneous flap or iliac crest osteomuscular flap is used for bone and intraoral reconstruction and a soft tissue free flap such as an anterolateral thigh, rectus abdominis, or radial forearm flap is used for external coverage. 9 10

Anterior mandibular defects require some form of skeletal reconstruction, as opposed to lateral defects, which can have reasonable outcomes with soft tissue reconstruction only. 11 This is ideally performed with vascularized bone. Bridging reconstruction plates, while reasonable for lateral bony defects, are associated with a high rate of complications in anterior defects. 12 13 The plates are covered with soft tissue, either as pedicled or free flaps. The most common complication of bridging plates is extrusion. 14 Intraoral exposures are thought to occur due to attenuation of the overlying tissue from muscle denervation, lip ptosis, and the cheese-cutter effect of a plate on overlying tissues. 13 Cutaneous extrusion occurs due to pressure necrosis of the overlying skin. Pedicled flaps, compared with free flaps, have greater tension pulling on the flap, and therefore, plate extrusion is more likely. 15 Mandibular reconstruction with bone has been shown to have superior functional outcomes compared with bridging plates with regard to oral competence, speech, and diet. 16 Vascularized bone flaps are the gold standard method for mandible reconstruction. The fibula is our flap of choice because of the ability to perform multiple osteotomies, good bone stock for dental implants, and acceptable donor site morbidity. The skin paddle can resurface moderate-sized defects. Large through-and-through defects involving the entire lip and anterior floor mouth have soft tissue requirements that far exceed the cutaneous territory of the peroneal artery perforators, and therefore require a second soft tissue flap. A simple way to accomplish this is by using a regional pedicled flap, such as a pectoralis major musculocutaneous flap or a supraclavicular flap. Although this obviates a second set of microvascular anastomoses, it limits the freedom of inset and the available volume of soft tissue. A second soft tissue free flap allows a more anatomic reconstruction in terms of size, tissue thickness, and freedom of inset. For extensive composite anterior mandible defects, we use a three-segment fibula bone for anterior arch reconstruction and its skin paddle for intraoral resurfacing. External soft tissue defects are usually extensive and comprises the lips, chin, and neck. The anterolateral thigh (ALT) flap is the tissue of choice, because it provides a large volume of thin tissue. One patient in our series had a dominant rectus perforator; therefore, the anteromedial thigh perforator flap was harvested. In addition, both ALT flaps were previously used; therefore, a vertical rectus abdominis musculocutaneous flap was used. Flaps should be of adequate size. If a flap smaller than the defect is used, it will distort the anatomy, as it contracts and can downgrade functional outcomes. We recommend suspension of the reconstructed lower lip to help maintain oral continence and create a lower labial sulcus.

A few important points merit special mention.

First, preoperative planning is critical. A computed tomogram is extremely useful for identifying the position of the remaining mandible. The lateral mandibular segments are typically pulled medially by the mylohyoid muscles and superiorly by the masseter, temporalis, and medial pterygoid muscles. 17 Nutritional evaluation is important because many of these patients are nutritionally deficient due to difficulty with oral intake. Patients may require feeding tubes for preoperative nutritional optimization.

Second, with delayed reconstruction, it is essential to recreate the original defect by radical excision of the scar tissue and release of contractures. Only then can the extent of bone and soft tissue required for reconstruction be determined. A chronically contracted wound distorts the anatomy and masks the extent of soft tissue deficiency. Soft tissue and residual native mandible segments must be restored to their correct positions.

Third, recipient vessels may have been depleted in previous reconstructive attempts. Every attempt is made to use separate vessels for each flap. Chain linking a second flap on the distal runoff of the first flap has been shown to have higher rates of partial and total flap loss. 18 19 This is thought to be due to the geometry of the arrangement leading to pedicle compression or kinking and thrombogenicity of the first anastomosis and steal phenomenon. 19 20 Even in a vessel-depleted neck, the transverse cervical vessels and the terminal branches of the external carotid artery are usually available.

Finally, revisions are frequently required for flap resuspension, debulking, or lipofilling. Regardless of the initial reconstruction, patients nearly always require several revision procedures, because it is almost impossible to achieve an ideal outcome in a single operation.

This study has several strengths and weaknesses. This is a small series of patients, as such severe secondary deformities are rare. The major strength of this study is the objective assessment of functional outcomes. Although these cases are challenging, our experience shows that adherence to the fundamental reconstructive surgery principles of recreation of the original defect and anatomic restoration of structures can result in the successful rehabilitation of these patients.

### Conclusion

Salvage reconstruction of established composite anterior mandible defects is challenging because of distortion of the normal lower facial anatomy by scar contracture of unsupported soft tissues. The keys to successful reconstruction are recreation of the original defect, use of two free flaps for optimal volume replacement, anastomoses to separate sets of healthy vessels, and suspension of the lip. Revisions are required to maximize the aesthetic and functional outcomes.
